# Redundant Mechanisms Recruit Actin into the Contractile Ring in Silkworm Spermatocytes

**DOI:** 10.1371/journal.pbio.0060209

**Published:** 2008-09-02

**Authors:** Wei Chen, Margit Foss, Kuo-Fu Tseng, Dahong Zhang

**Affiliations:** 1 Department of Zoology, Oregon State University, Corvallis, Oregon, United States of America; 2 Molecular and Cellular Biology Program, Oregon State University, Corvallis, Oregon, United States of America; 3 Center for Genome Research and Biocomputing (CGRB), Oregon State University, Corvallis, Oregon, United States of America; Adolf-Butenandt-Institut, Germany

## Abstract

Cytokinesis is powered by the contraction of actomyosin filaments within the newly assembled contractile ring. Microtubules are a spindle component that is essential for the induction of cytokinesis. This induction could use central spindle and/or astral microtubules to stimulate cortical contraction around the spindle equator (equatorial stimulation). Alternatively, or in addition, induction could rely on astral microtubules to relax the polar cortex (polar relaxation). To investigate the relationship between microtubules, cortical stiffness, and contractile ring assembly, we used different configurations of microtubules to manipulate the distribution of actin in living silkworm spermatocytes. Mechanically repositioned, noninterdigitating microtubules can induce redistribution of actin at any region of the cortex by locally excluding cortical actin filaments. This cortical flow of actin promotes regional relaxation while increasing tension elsewhere (normally at the equatorial cortex). In contrast, repositioned interdigitating microtubule bundles use a novel mechanism to induce local stimulation of contractility anywhere within the cortex; at the antiparallel plus ends of central spindle microtubules, actin aggregates are rapidly assembled de novo and transported laterally to the equatorial cortex. Relaxation depends on microtubule dynamics but not on RhoA activity, whereas stimulation depends on RhoA activity but is largely independent of microtubule dynamics. We conclude that polar relaxation and equatorial stimulation mechanisms redundantly supply actin for contractile ring assembly, thus increasing the fidelity of cleavage.

## Author Summary

In animal cells, the last step of cell division, or cytokinesis, requires the action of a contractile ring—composed largely of actin and myosin filaments—that cleaves the cell in two. Before the cell divides, it first duplicates its genome and separates the chromosomes into the two newly forming daughter cells, a task carried out by a structure called the spindle apparatus, which is composed mostly of long polymers called microtubules. The site of cleavage must occur between the segregating chromosomes—at the spindle equator—to ensure that each cell receives the proper number of chromosomes. In addition to separating the chromosomes, microtubules are also essential for inducing cytokinesis—but how they do this is controversial. For example, the “polar relaxation” hypothesis proposes that the astral microtubules, which radiate outward, cause contractile elements to flow from the polar cortex toward the equator, resulting in furrowing. In contrast, the “equatorial stimulation” hypothesis proposes that the spindle microtubules directly stimulate cleavage exclusively at the equator. Using a novel approach, we demonstrate that both mechanisms are in fact functioning together to recruit actin filaments to the nascent ring, providing redundancy that increases fidelity. Specifically, we were able to mechanically alter the distribution of actin filaments in living, dividing cells by using a microscopic needle to manipulate microtubules while perturbing the cytoskeleton with chemical compounds. Our high-resolution microscopy data advance the understanding of both proposed mechanisms. We also documented a novel, microtubule-based mechanism for transporting actin aggregates to the equatorial cortex. These results help to resolve a long-standing dispute concerning this fundamental cellular process.

## Abstract 3

How is actin recruited to assemble a contractile ring during cytokinesis? Combining micromanipulation with pharmacological perturbation, this comprehensive study elegantly documents the contributions of two complementary mechanisms within one cell.

## Introduction

The contractile ring is a dynamic, actomyosin-based structure whose constriction generates a cleavage furrow, ultimately producing two daughter cells. To ensure accurate distribution of chromosomes to each cell, the location of the division plane and the timing of cleavage must be tightly regulated to coordinate with chromosomal segregation. In animal cells, this coordination is mediated by the spindle apparatus, which both segregates the chromosomes during anaphase and signals the cortex as to where and when the contractile ring should be assembled (reviewed in, e.g., [1–4]). In addition to its structural components, the ring contains numerous other proteins, some of which are thought to recruit or retain ring components, while others function as regulatory or signaling molecules. For example, the GTPase RhoA and its guanine nucleotide exchange factor help regulate polymerization of actin and myosin within the ring, thus promoting its constriction (reviewed in [5]). Some proteins are localized both to the central spindle and the equatorial cortex in a temporally dynamic manner. It is not known whether these proteins represent independent pools or whether the proteins diffuse or are somehow transported between locations.

Spindle microtubules are known to play a critical role in specifying the cleavage plane in animal cells. Evidence from divergent organisms shows that the cleavage furrow can be initiated at the spindle equator by astral microtubules, central spindle microtubules, or both (reviewed in [1]). Furthermore, microtubules have been shown to be the only structural constituent of the spindle apparatus required for furrow induction, regardless of their original location within the spindle [6]. However, it remains unclear whether microtubules induce furrow formation by mechanisms of polar relaxation and/or equatorial stimulation of the cell cortex (reviewed in [1–4,7]).

The polar relaxation model, as originally proposed by Wolpert [8], postulates that inhibitory signals are sent from astral microtubules to the polar cortex to decrease cortical tension locally. Computer simulations indicate that high microtubule density at the poles may produce the highest surface tension at the equator, where the contractile ring forms [9,10]. In sea urchin eggs, just before cytokinesis, tensile forces become elevated globally, and thus relaxation of tension at the poles may lead to furrow formation at the equator [11,12]. Furthermore, in cells with depolymerized or genetically shortened microtubules, the overall cortical contractility is higher, albeit disorganized (e.g., [13–15]). In theory, microtubules could induce polar relaxation by a number of different mechanisms. Microtubules could inhibit assembly (or promote disassembly) of polar actin filaments. Alternatively, they could inhibit contractile activity of the polar filaments. For example, microtubules might inhibit Rho-dependent actin contractility through localized sequestration and inhibition of GEF-H1, an activator of Rho [16]. Or instead, microtubules could induce polar relaxation by altering the distribution of cortical actin filaments, relocating them away from the poles in a process known as cortical actin flow. Cortical flow of actin has been observed in cultured mammalian cells [17].

The equatorial stimulation model proposes that furrowing is induced by stimulatory signals sent from the central spindle midzone and/or the spindle asters to the equatorial cortex. The classic “torus experiment” by Rappaport [18] demonstrated that astral microtubules from two opposing asters, not connected by a spindle, are sufficient to induce ectopic furrow formation in echinoderm embryos. Because the cell was topologically manipulated to eliminate the poles, the implication was that polar relaxation could not be invoked as the mechanism for furrowing—although technically, a role for cortical flow has not been excluded. Similarly, astral microtubules from two neighboring spindles can define such an ectopic furrow in a fused epithelial cell [19]. For monopolar spindles, it was proposed that furrow formation was stimulated by a subset of astral microtubules stabilized by chromosomes [20], or by bundles of central spindle-like microtubules [21]. In cultured rat cells, when communication between the central spindle and equatorial cortex is disrupted by a small “perforation” (i.e., a deep indentation of the membrane, located peripherally within the equatorial plane), a furrow fails to form at the equatorial cortex [22]; the furrow initiates instead at the perforation site—as if the central spindle were now signaling to an “interior cortex” located on the spindle side of the perforation. Proteins localized to the central spindle, such as centralspindlin (consisting of a RhoGAP and a kinesin) and RhoGEF, are important for organizing midzone microtubules and signaling the equatorial cortex for furrow induction [5,23,24]. Recently, LET-99 and heterotrimeric G proteins were shown to play important roles in aster-positioned cytokinesis in Caenorhabditis elegans embryos [25]. In C. elegans zygotes, stimulatory signals from asters and the spindle midzone are both important for cytokinesis [26–28]. In *Drosophila*, it was very recently shown that F-actin–associated vesicles co-localize with central spindle and peripheral microtubules. Thus, it was hypothesized that the central spindle may somehow coordinate co-transportation of actin and new membrane to the ingressing cleavage furrow [29].

Although it has not been directly demonstrated that polar relaxation and equatorial stimulation coexist in a single cell during cytokinesis, other groups have worked to document the relationship between astral-derived and central spindle-derived cytokinetic signals. For example, in the Drosophila asterless mutant, a suboptimal version of cytokinesis can occur despite the absence of asters from the male meiotic spindle, demonstrating that astral microtubules are dispensable for inducing cytokinesis in male meiosis [30]. Bringmann and Hyman showed that astral and central spindle signals both help position the cytokinetic furrow during the first zygotic division in C. elegans [26]; however, cortical tension was not specifically addressed. The astral signal in C. elegans was later shown to be distinct from the central spindle signal and to negatively regulate distribution of cortical myosin [28]. In contrast, myosin II in cultured mammalian cells does not undergo cortical flow, as determined by kymography, whereas actin does [17]. Furthermore, the authors postulated that cortical actin is not the sole source of equatorial actin [17]; the other source was not identified. Hu et al. recently observed HeLa cells undergoing drug-induced monopolar cytokinesis. They postulated that feedback loops (i.e., bi-directional signaling) between midzone-like spindle microtubules and various furrow components could promote polarization in these cells, and also that cortical flow could help organize microtubules [21]. Our work builds on, complements, and extends these ideas, using a novel experimental approach.

If we assume that polar relaxation and equatorial stimulation can coexist in cytokinesis, it is not obvious how microtubules manage to provide opposing signals in the same cell, i.e., to relax the polar cortex yet stimulate the equatorial cortex. To address this question, and to probe the mechanistic link between cortical properties and contractile ring assembly, we used fluorescently labeled cytokinetic cells of the silkworm Bombyx mori. These cells allowed us to examine the distribution of actin filaments as driven by micromanipulated microtubules, with or without drug treatments that affect either microtubule dynamics or actin assembly. We show that loss of cortical actin may occur at any region of the cell cortex adjacent to dynamic microtubules, due to cortical flow. Stimulation, as gauged by rapid de novo assembly and delivery of actin aggregates, may also occur at any region of the cell cortex, mediated by the bundled, overlapping plus ends of central spindle microtubules. Our data show that polar relaxation and equatorial stimulation mechanisms coexist in silkworm spermatocytes and are compatible with dual roles for microtubules. Furthermore, our work provides mechanistic advances and demonstrates how both mechanisms can independently supply actin for contractile ring assembly.

## Results

### The Spindle Apparatus and Cytokinesis in Spermatocytes of the Silkworm B. mori

**Figure 1:**
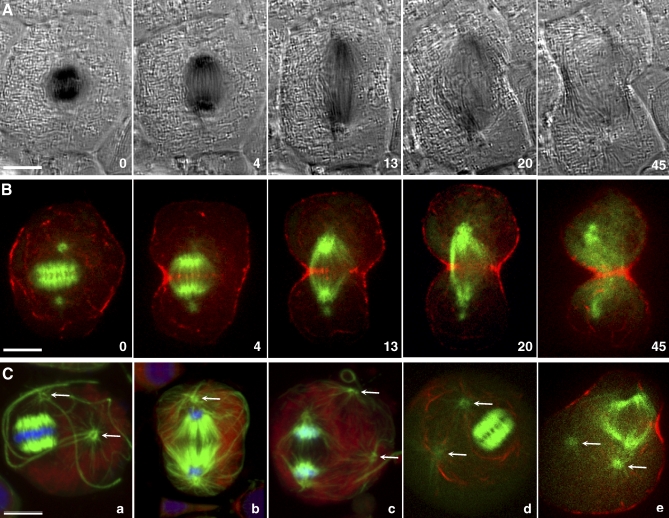
Cytokinesis of Silkworm Spermatocytes (A) Polarization micrograph of a dividing spermatocyte. (B) Confocal micrographs of a spermatocyte microinjected with rhodamine tubulin (microtubules false colored green), and low-level Alexa Fluor 488 phalloidin (actin false colored red). Actin aggregates appeared at the equatorial microtubule plus ends during early anaphase (0–20), then fused into a contractile ring that bisected the cell (4–45). (C) Astral flagella, as well as naturally, asymmetrically positioned asters, were visible in dividing spermatocytes. Astral flagella were visible only in fixed, immunostained cells (a–c). Astral microtubules were prominent in fixed anaphase spermatocytes (e.g., b). One aster was loosely attached to the spindle (arrow). Occasionally, the asters (arrows) in fixed (a and c) and live (d and e) spermatocytes (arrows) were naturally positioned on the same side of the spindle. a and d, metaphase; b, c and e. anaphase. Fixed cells were double-immunolabeled for actin and tubulin; live cells were labeled as in (B). Time in min. Time 0 in (A and B), anaphase onset. Bars, 10 μm.

Spermatocytes cultured underneath a layer of halocarbon oil were relatively large (∼33 μm in diameter), optically clear, and amenable to micromanipulation. Digital-enhanced polarization microscopy on cells from late metaphase to early anaphase revealed a short but robust spindle (Figure 1A and Video S1; *n* = 9), whose relatively small size provided ample space for manipulation of spindle microtubules. To visualize the dynamics of microtubules and actin filaments during cytokinesis, we microinjected metaphase-stage spermatocytes with rhodamine tubulin and low-level Alexa Fluor 488 phalloidin (Figure 1B and Video S2; *n* = 4). The fluorescently labeled cytoskeleton was imaged using spinning disc confocal microscopy. Furrow initiation occurred during early anaphase after actin aggregates emerged at the interdigitating microtubule plus ends at the equator (Figure 1B, between 0 and 4). The aggregates gradually enlarged before coalescing with cortical actin filaments to assemble the contractile ring that constricts the cell (Figure 1B, 4–45). The time from anaphase onset to cleavage was not significantly different for the uninjected control cells (48.4 ± 2.5 min) and the injected, double-labeled cells (49.5 ± 5.0 min), suggesting that the low-level phallodin was not perturbing actin function.

Image stacks of fixed, immunostained cells revealed dense arrays of astral microtubules radiating from the centrosomes to the polar cortex (Figure 1C, a–c; *n* = 15). Notably, asters in both fixed (Figure 1C, a–c) and live (Figure 1C, d and e; *n* = 15; also in Figure 1B, 0) cells appeared to be attached only loosely to the spindle, which is a natural phenomenon in silkworm spermatocytes [31]. Presumably due to the centrioles' motile flagellar axonemes (Figure 1C, a–c; as seen in immunostained cells), both asters were mobile within the cell. In approximately 15% of the cells, the asters even moved to the same side or the same pole of the spindle, as documented in metaphase (Figure 1C, a and d) and anaphase (Figure 1C, c and e) stage cells. This natural phenomenon makes silkworm spermatocytes an ideal system for separating the role of asters from that of the central spindle in cytokinesis.

### Evidence for Polar Relaxation as Gauged by Redistribution of Cortical Actin

#### Cortical flow of actin filaments driven by spindle microtubules.

**Figure 2:**
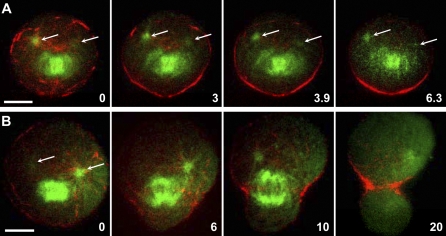
Cortical Actin Filaments Were Excluded from the Polar Cortex Bordering the Asymmetrically Distributed Asters (A) Both asters (arrows) were naturally positioned at the upper pole of the spindle. Cortical actin filaments flowed towards the lower polar cortex during early anaphase. Cells in Figure 2 were labeled as in Figure 1B. Time 0 depicts anaphase onset. (B) Cortical actin filaments, excluded by asymmetrically distributed asters (arrows), assembled a contractile ring around the equator of the naturally shifted spindle. Actin aggregates were also present by later anaphase. Time in min. Time 0, anaphase onset. Bars, 10 μm.

In cells in which both asters (Figure 2A, arrows) happened to be located at the same pole of the spindle, cortical actin filaments appeared to flow to the opposite side of the cell during anaphase (Figure 2A, 0–6.3, and Video S3; *n* = 4. Note: panels were chosen to highlight cortical flow; these focal planes are not optimal for viewing actin aggregates). In such cells, the midzone of the central spindle was never aligned with the cell's equator, and these cells divided asymmetrically around the equator of the shifted spindle (Figure 2B and Video S4; *n* = 6). Our observation suggested that astral microtubules could relax the polar cortex by excluding cortical actin filaments from the pole. However, since the asymmetric furrow was aligned with the spindle equator, it was also possible that the spindle midzone signaled the band of cortex that encircled the spindle equator, stimulating recruitment of actin to the furrow. To discern between these alternatives, we needed to assess whether actin could be redistributed within the cortex in the absence of an intact central spindle. Thus, we used a microneedle to push the spindle poles together in anaphase, mechanically remodeling the spindle apparatus. We refer to this operation as “collapsing” the spindle. During the remodeling process, the spindle was simultaneously relocated to a region of the cell previously unexposed to any furrow cues (e.g., nascent actin aggregates). By collapsing the spindle, we accomplished three objectives. First, the act of collapsing served to reorganize the central spindle, destroying its equatorial configuration. This likely impeded its ability to stimulate the cortex in its new location, preventing the formation of a complete, circular furrow. Second, relocation of the spindle presumably prevented deposition of additional furrow cues at the original cortical site, and depending on the timing of the move, may well have prevented deposition of any cues at the original location. Third, relocation and collapsing of the spindle allowed us to induce controlled exclusion of actin filaments from a region of our choice; relocation brought the spindle close enough to the cortex to initiate cortical flow, whereas collapsing exposed or freed some microtubule plus ends that originally interdigitated with the opposite half of the spindle, potentially altering their dynamic properties (see below).

**Figure 3:**
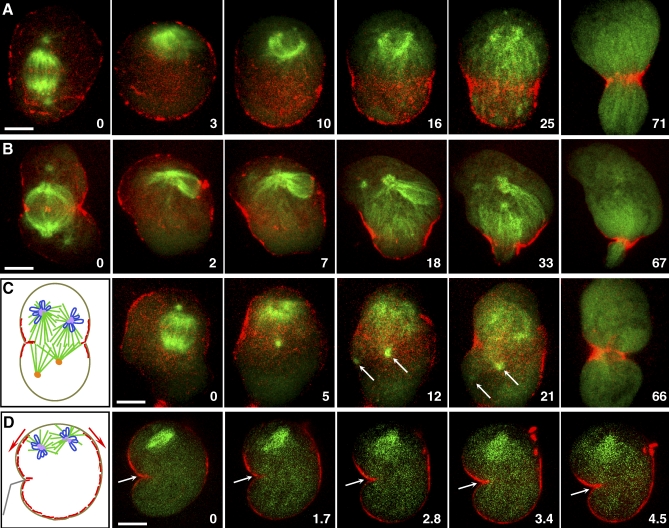
Cortical Flow of Actin Filaments Was Driven by Spindle Microtubules (A) During anaphase (0), when the spindle apparatus was collapsed, and pushed with a microneedle to an arbitrary region of the cell cortex (3), actin filaments flowed to the opposite side of the cell (3–25). This redistribution of actin filaments by spindle microtubules resulted in asymmetric cell division (71). (B) Repositioning and reorganization of the spindle (2) during telophase resulted in a similar scenario, preceded by regression of the original furrow. (C) An anaphase cell (with only one aster visible in this focal plane; 0) was mechanically remodeled by moving spindle microtubules to the upper portion of the cell and collapsing the spindle (shown at top of cell, 5–21). The collapsed spindle was manipulated to detach and relocate first one of its asters (dot in center of cell, 5) and then its second aster (to left of first aster; both asters marked by arrows, 12–21). Possibly due to actin exclusion (5–21) by microtubules from both structures, the contractile ring formed between the collapsed spindle and the pair of detached asters (21–66). This is shown in the schematic diagram (representing the cell in 21). (D) A schematic of the set-up, with fluorescence images of microtubule-driven actin flow blocked by a microneedle. The schematic shows the cell's outline, the location of the needle (gray), and the direction of flow of cortical actin (red), as induced by the collapsed, repositioned spindle (green, at top). The manipulation needle (arrows), which indented but did not pierce the plasma membrane, locally intercepted the actin flow; brighter fluorescence accumulated on the side of the needle that faced the repositioned spindle (1.7–4.5). The three large red objects (3.4–4.5, upper right) are cell division scars, i.e., remnants from former cytokinetic rings. These scar structures can also move around within the cortex during cortical flow. Note: despite removal of the holding needle, the spindle is not depicted as a giant aster, since the reorganization from monopolar spindle to aster takes at least several minutes. Cells in (A–D) were labeled as in Figure 1B. Time in min. Time 0 in (A), (C), and (D), mid to late anaphase; in (B), telophase. Bars, 10 μm.

Shortly after anaphase onset (Figure 3A, 0, and Video S5; *n* = 12), we pushed the spindle apparatus with a microneedle to an arbitrary region of the cell cortex while collapsing it as described (Figure 3A, 3). Shortly thereafter, cortical actin filaments began to flow away from the spindle microtubules to the opposite side of the cell (Figure 3A, 10). Because actin filaments are a major component of actomyosin contractile elements, their exclusion from the cortex near the spindle would presumably result in cortical relaxation. This microtubule-driven actin flow resulted in asymmetric distribution of cortical actin filaments (Figure 3A, 10) and initiation of contractile ring assembly (Figure 3A, 16, and Video S5) around the presumptive, newly defined zone of microtubule overlap [32]. The microtubule-driven actin flow persisted into telophase, as shown in similar experiments in which cells were manipulated while undergoing cytokinesis (Figure 3B and Video S6; *n* = 26). This resulted in asymmetric placement of the contractile ring (Figure 3B, 18–37). Because randomly dislocated asters (Figure 2) and arbitrarily repositioned, collapsed spindles (Figure 3A and 3B) caused cortical actin flow, we hypothesized that cortical relaxation could be induced at any region of the cell cortex by microtubules from any source. To test this idea, we mechanically remodeled cells by placing both asters in one region of the cell, separated from the asterless, collapsed spindle, as depicted in the schematic in Figure 3C (Figure 3C, 5–12, and Video S7; *n* = 8). As expected, the contractile ring assembled between the two noninterdigitating structures due to actin exclusion from both “poles” (Figure 3C, 5–66, and Video S7).

#### Microtubule-driven cortical actin flow could be intercepted by a microneedle.

From the experiments shown in Figure 2 and Figure 3A–3C, it appeared that cortical actin filaments were redistributed to the equatorial cortex by astral microtubules (or by those in a collapsed spindle), rather than assembled there, de novo. To confirm this, we decided to use a microneedle to indent a region of the cortex, distant from the presumptive source of actin. We predicted the needle would intercept part of the actin flow, resulting in the accumulation of cortical actin filaments on the side of the needle-indented cortex that faced the source of actin, i.e., the side closer to the repositioned spindle (Figure 3D, diagram). We induced microtubule-driven cortical actin flow in a microinjected anaphase cell (Figure 3D, 0, and Video S8; *n* = 5), and then indented but did not penetrate the cell membrane with the tip of a microneedle (Figure 3D, arrows; not observable in fluorescence channels). As predicted, the needle regionally blocked actin flow, with actin fluorescence becoming increasingly brighter on the side of the needle facing the repositioned, collapsed spindle (Figure 3D, 1.7–4.5, and Video S8). As an internal control, no obstruction of actin flow was observed at the unblocked side of the cell cortex. This experiment substantiated our idea that spindle microtubules could exclude cortical actin filaments during anaphase and early telophase, thus presumably relaxing the cell cortex they contacted.

#### Cortical stiffness correlated with local density of cortical actin.

**Figure 4:**
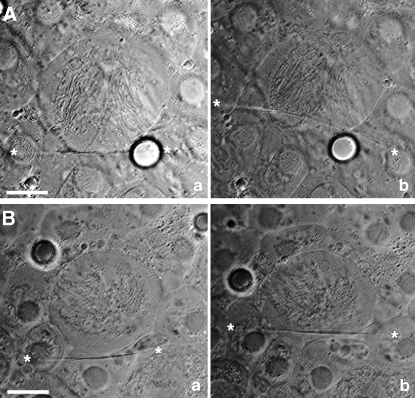
Cortical Stiffness Correlates with Local Density of Cortical Actin (A) After the spindle was repositioned and collapsed to induce cortical flow, we tested the stiffness of the actin-dense region of the cortex. (a) The needle (marked with asterisks) in its initial position, tangential to the actin-rich cortex. (b) After the needle was pushed against the cortex, the stiff cortex deflected the needle. (B) After rotating the cell in (A) by 180°, the cortical stiffness of the actin-depleted region of the cortex was tested. (a) The needle (marked with asterisks) in its initial position, tangential to actin-depleted cortex. (b) After the needle was pushed against the cortex, the relaxed cortex was deformed by the needle without deflecting it. F-actin was labeled using low-level Alexa Fluor 488 phalloidin to monitor flow. Bars, 10 μm.

We had been using local density of cortical actin to infer cortical stiffness, and we needed to test the validity of this assumption. To assay the degree of cortical stiffness in a region of high actin density relative to one of low actin density, cortical flow of actin filaments was induced using a repositioned, collapsed spindle to generate a density differential. After cortical flow was induced, the cortex farthest from the spindle (high actin density) was probed by depression with the side of a flexible microneedle. As predicted, the needle tip was bent by the cortex (Figure 4; *n* = 7). When the cortex on the opposite side of the cell (low actin density) was probed using the same needle, the cortex was deformed, while the needle remained unbent (Figure 4; same seven cells). (Similar results were obtained using grasshopper spermatocytes; unpublished data (*n* = 6).) Thus, we concluded that regions with a high density of cortical actin are indeed stiffer (i.e., less relaxed) than regions of low density, justifying our use of the term “polar relaxation” to describe redistribution of actin via cortical flow.

### Evidence for Equatorial Stimulation as Gauged by De Novo Assembly of Actin

#### De novo assembly and delivery of actin aggregates mediated by microtubule plus ends.

**Figure 5:**
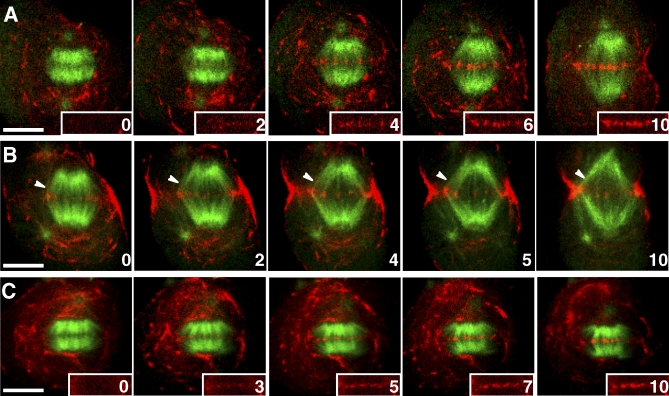
Interdigitating Microtubule Plus Ends Hosted De Novo Actin Assembly and Delivered Actin Aggregates to Equator (A) Emergence (0–2) and growth (2–10) of nascent actin fluorescence at the equatorial microtubule plus-end overlap region reflect de novo assembly of actin aggregates. (B) A splaying microtubule bundle (arrowheads) delivered the actin aggregate into the ingressing furrow. Cells in (A) and (B) were labeled as in Figure 1B. (C) De novo assembly of actin aggregates was not inhibited by microtubule stabilization (compare to (A) and Figure 1B). Microtubules were labeled and stabilized by Oregon green paclitaxel, and actin filaments were labeled by rhodamine phalloidin. Insets: actin channel. Time in min. Time 0 in (A and C), immediately before anaphase onset; in (B), late anaphase. Bars, 10 μm.

Two sources of actin may contribute to contractile ring assembly: pre-existing actin filaments from the cell cortex and the cytoplasm, and actin aggregates that are assembled de novo at the equator. We have demonstrated that microtubule-driven cortical flow provides a mechanism by which cortical actin is excluded from the polar cortex. (Polar microtubules may also play a role in clearing cytoplasmic actin filaments from the polar region. See legend to Video S4.) We hypothesized that microtubules might stimulate de novo actin assembly at the equator, since the speckles of actin fluorescence that were detected at equatorial microtubule plus ends increased in number and size as anaphase progressed (Figure 1B). To detect de novo actin assembly, we monitored its dynamics starting at the metaphase-anaphase transition in cells with labeled microtubules and actin filaments (Figure 5A and Video S9; *n* = 15). Before onset of anaphase, actin fluorescence was absent from the spindle equator where the aligned metaphase chromosomes were located (Figure 5A, 0). As the cell entered anaphase (Figure 5A, 2), speckles of actin fluorescence soon emerged at the spindle midzone where microtubule plus ends overlapped (Figure 5A, 2–4). The speckles gradually grew into bigger aggregates as anaphase proceeded (Figure 5A, 4–10, and Video S9). The de novo emergence and accumulation of actin fluorescence was readily apparent when actin alone was visualized, using the rhodamine channel (Figure 5A, insets). Notably, nascent actin aggregates were assembled across the entire midzone of the central spindle, and delivered laterally to the incipient contractile ring by microtubules (Figure 5B and Video S10; *n* = 13). This novel means of delivery was driven by a structural reorganization of each spindle half: microtubules became more focused at their minus ends (at the spindle poles), while splaying out toward the equatorial cortex at their plus ends, thus enlarging the spindle midzone region. These splaying microtubules were organized into bundles, some of which were coated with an actin aggregate at the plus ends of the bundle, enabling its delivery to the cortex. Lateral transport appeared to begin around the time of furrow initiation, while cortical flow was already in progress. However, we could not definitively rule out an earlier starting point, since small numbers of peripheral microtubules could have escaped detection. Moreover, it was problematic to define a precise endpoint, as the trajectory of the outwardly splaying spindle was confounded by the ingressing furrow.

### Polar Relaxation Was Dependent on Microtubule Dynamics, whereas Equatorial Stimulation Was Largely Independent

#### De novo actin assembly was independent of microtubule dynamics.

Because microtubules were involved in both actin exclusion from the polar region and de novo actin assembly at the equator, we asked whether either process required dynamic microtubules. To address this question, we repeated the de novo actin assembly experiments (as in Figure 5A) in cells whose microtubules were simultaneously labeled and stabilized with Oregon green paclitaxel (Figure 5C and Video S11; *n* = 11). We determined whether paclitaxel-treated cells had entered anaphase by gently probing the chromosomes with a microneedle—allowing us to assess whether homologous chromosomes were physically separable or still attached to one another. Following the onset of anaphase, chromosomes in spindles that were stabilized at the metaphase-anaphase transition could be separated from their homolog, but they could not move poleward (unpublished data). However, de novo actin assembly at the midzone of microtubule plus ends was apparent (Figure 5C, 3–10; insets show the actin channel alone; and Video S11). Emergence and accumulation of actin fluorescence at the spindle midzone appeared similar in stabilized and nonstabilized cells (compare to Figure 5A), demonstrating that aggregate assembly was independent of microtubule dynamics. However, the assembled actin aggregates remained at the midzone of the spindle stabilized at the metaphase-anaphase transition. This failure of delivery suggests that at later stages, when the central spindle is remodeled to produce the polar focusing of microtubules and the splaying of bundles, dynamic microtubules may be required.

#### Lateral transport of de novo–assembled actin aggregates by paclitaxel-stabilized microtubules.

**Figure 6:**
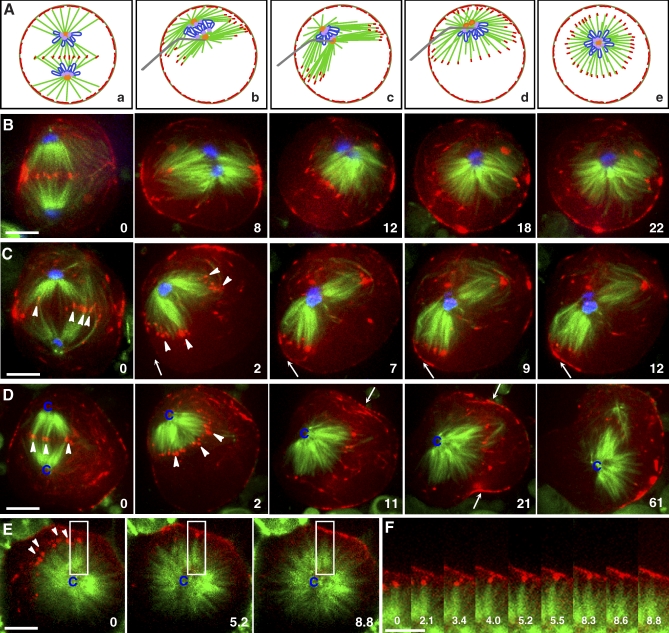
Redistribution of Cortical Actin, but Not Equatorial Stimulation, Was Dependent on Microtubule Dynamics (A) A schematic showing remodeling of the central spindle by micromanipulation. A post-anaphase spindle (a, green) was collapsed using a micromanipulation needle (b, gray). The manipulation created two lateral microtubule bundles, with the exposed microtubule plus ends of the bundles pointing toward opposite sides of the cortex. The interior ends of the bundles flanked chromosomes (c, blue), kinetochores (c, purple) and centrosomes (c, orange). If the collapsed spindle was brought close to the cortex and held in place by a microneedle, it reorganized into a monopolar spindle (d). If the holding needle was removed, the spindle usually reorganized into a giant aster (e). (B) Actin exclusion was inhibited when the remodeled spindle was stabilized by paclitaxel. Neither the stabilized lateral spindle (8) nor the evolving monopolar spindle (12–22) induced unidirectional flow of actin filaments (compare to Figure 3). Cells in (B–F) were labeled as in Figure 5C. (C) Actin aggregates were assembled at the plus ends of stabilized, laterally oriented microtubule bundles (0–2, arrowheads), and were delivered to the non-equatorial cortex where splaying microtubule bundles were in contact with the cortex (2–12, arrows). (D) A monopolar spindle (2) gradually splayed its microtubule bundles toward the cell cortex (2–21), which delivered actin aggregates (2, arrowheads) and induced a furrow (11–21, arrows). The furrow eventually regressed (61). (E) Tracking of an actin aggregate delivered from the plus ends of one microtubule bundle to the cell cortex. The giant aster had chromosomes in its center and microtubule plus ends pointing outward, as in (A, panel e). When the aster was placed near the cell cortex using a microneedle, an actin aggregate moved away from the microtubule bundle plus ends and merged into the cortex (box, 0–8.8). (F) The region of interest in (E) is shown with additional intervening time points to highlight the increasing cortical fluorescence caused by merging of actin aggregates. Time in min. Time 0 in (B), (D), and (E), mid to late anaphase; in (C), telophase; in (F), identical to 0 in (E). Bars, 10 μm.

To further test how central spindle microtubules deliver de novo–assembled actin aggregates to the cell cortex, we remodeled these actin-tipped microtubules with a microneedle to expose their plus ends to the cortex (Figure 6). After anaphase onset, a central spindle (Figure 6A, a) was collapsed by pushing the spindle poles together (Figure 6A, b). Microtubules from the collapsed spindle reorganized into two lateral bundles that extended from the centrosomes outward, toward opposite sides of the cell cortex, with each bundle containing microtubules from both original half spindles (Figure 6A, c). Over time, the lateral spindle could reorganize into a monopolar spindle (Figure 6A, d) if the chromosomes and centrosomes were held together with a microneedle, or into a giant aster, if the needle was removed (Figure 6A, e). To prevent the formation of new regions of microtubule overlap [32], and to investigate dependence on dynamics, we stabilized microtubules with Oregon green paclitaxel (Figure 6B, *n* = 4 slowly evolving spindles; 6C, *n* = 12 lateral spindles; 6D, *n* = 6 monopolar spindles; and 6E and 6F, *n* = 7 giant asters).

We mechanically positioned the remodeled and stabilized spindle so that its exposed microtubule plus ends, which were coated with the de novo–assembled actin aggregates, were removed from the original equatorial cortex and thus from any cues already present there. In all spindle configurations tested (Figure 6A, b–e), actin aggregates were delivered by the bundled microtubules to the nearby cortex. Notably, microtubule splay in a lateral spindle gradually brought microtubule plus ends close to the cell cortex, and hence allowed the delivery of actin aggregates (Figure 6C and Video S13). Furrow initiation could occur in such manipulated cells, provided the actin aggregates were delivered to a cortical zone that encircled the cell (Figure 6D, arrows). Using time-lapse imaging, we followed a giant aster positioned near the cell periphery (viewed from above, as in Figure 6A, e) and documented its delivery of an actin aggregate from the plus end of a microtubule bundle to the cell cortex (Figure 6E and 6F and Videos S14 and S15). The accumulation of actin aggregates made the cell cortex significantly brighter over time (Figure 6F, 0 onward), indicating that delivery of actin aggregates can proceed even in the absence of dynamic microtubules, so long as splay of central spindle microtubules is not blocked.

In the final stage of lateral transport, actin aggregates had to be released from the tips of the microtubule bundles to allow their delivery to the cortex. To examine this process in greater detail, the red and green channels were analyzed individually for signs of costaining at the tips of microtubule bundles (in transit from the central spindle to the cortex). The signal from the microtubules at the peripheral ends of the bundles was much weaker than the signal from the actin; thus, the costained regions appeared red rather than yellow in the merged images shown in Figure 6. However, close analysis revealed a correlation between the length of the costained region of the bundle and the distance from bundle tip to cortex (Table 1). This distance was actually measured from the distal end of the actin aggregate rather than from the distal end of the bundle itself. The length of the actin aggregate remained fairly constant—approximately 0.8 to 0.9 μm (Table 1). When a bundle was more than about 1.2 μm from the cortex, at least a portion of the aggregate (perhaps close to the entire aggregate) overlapped with the tip of the bundle. As the bundle, with its associated cargo, came within about 0.41–1.2 μm of the cortex, it was no longer possible for the entire length of the aggregate to fully overlap with the bundle, even taking into account the uncertainty of the measurements. In other words, the aggregate appeared to be “sliding” off the tip of the bundle, toward the cortex. Finally, as the actin-tipped bundle approached within 0.4 μm of the cortex, i.e., closer than the length of the aggregate itself, the aggregate was released completely from the bundle, and the overlap dropped to zero.

**Table 1:**
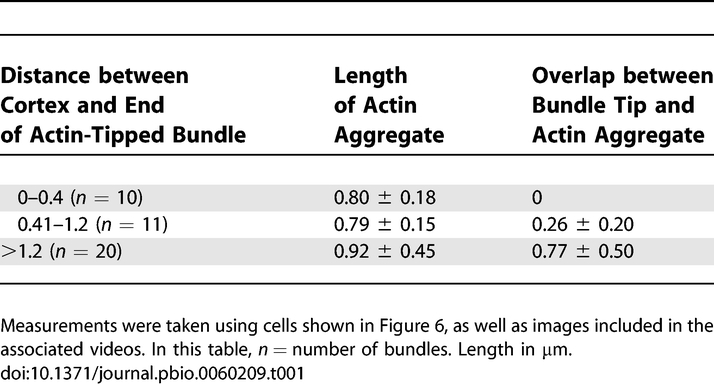
Measurements of Actin Aggregate-Tipped Single Bundles

#### Cortical actin flow was dependent on microtubule dynamics.

In contrast to the de novo assembly of actin aggregates, cortical flow of actin was highly dependent on dynamic microtubules. We attempted to induce cortical flow in paclitaxel-treated cells by collapsing the spindle with a microneedle while repositioning it close to the cortex (Figure 6B–6D, Video S12, and see also Figure 5C, 3–10). Cortical flow was clearly inhibited. This result supports our contention that cortical actin filaments are excluded from the polar region and redirected toward the equatorial cortex by dynamic microtubules.

### Equatorial Stimulation, but Not Polar Relaxation, Was Dependent on RhoA Activity

**Figure 7:**
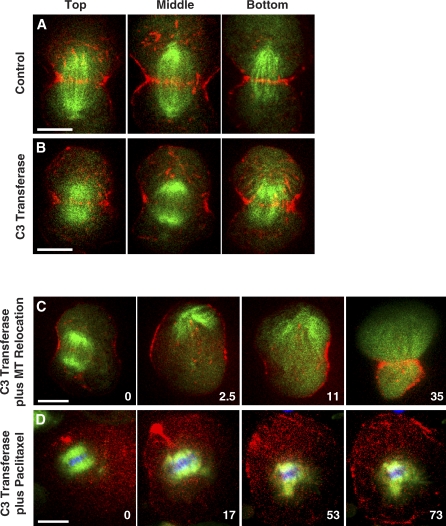
Equatorial Stimulation, but Not Redistribution of Cortical Actin, Was Dependent on RhoA Activity (A) A cytokinetic control cell, optically sectioned lengthwise, displayed actin aggregates at the microtubule plus ends of its spindle, both at the equatorial cortex (top and bottom) and in the central spindle (middle). Cells in (A and B) were labeled as in Figure 1B. (B) In a cytokinetic cell microinjected with C3 transferase to inhibit RhoA, actin aggregates at the microtubule plus ends of the central spindle were absent. (C) Despite inhibition of RhoA, actin flow was induced (2.5–11), following manipulation of the collapsed spindle to one side of the cell (2.5). Excluded filaments assembled into a contractile ring (35). Cell was labeled as in Figure 1B, and microinjected with C3 transferase. (D) In a cell treated with paclitaxel and C3 transferase, the furrow failed to initiate due to inhibition of both pathways for redistribution of actin. Actin filaments were scattered in the cytoplasm long after the cell entered anaphase, as determined by the presence of splaying microtubules (17–73). (D) was labeled as in Figure 5C. Time in min. Time 0 in (C), late anaphase; in (D), metaphase-anaphase transition. Bars, 10 μm.

We wanted to determine whether de novo assembly of actin aggregates at the plus ends of central spindle microtubules requires RhoA as a regulator. To do so, we microinjected late metaphase cells with C3 ribosyltransferase, a botulinum toxin that specifically inhibits Rho by ADP ribosylation [33]. We hypothesized that this inhibition would disrupt de novo actin assembly at the spindle midzone, without affecting cortical flow of actin. As expected, the C3 transferase-treated cell continued to divide but failed to accumulate actin aggregates in the central spindle (Figure 7). As seen in optical sections of control cells (*n* = 15), actin accumulated both at the equatorial cortex (Figure 7A, top and bottom) and at microtubule plus ends within the central spindle (Figure 7A, middle). In contrast, C3 transferase-treated cells (*n* = 9) accumulated actin at the equatorial cortex (Figure 7B, top and bottom), but not within the central spindle (Figure 7B, middle). This result implies that RhoA inactivation inhibits actin assembly at the plus ends of central spindle microtubules (i.e., the initial step of equatorial stimulation), but does not inhibit relocation of cortical actin from the poles to the equatorial cortex (i.e., polar relaxation).

Additional tests confirmed that in C3 transferase-treated cells, actin filaments were still excluded from the cortex adjacent to the dislocated spindle and incorporated into the contractile ring (Figure 7C, *n* = 8). However, when late metaphase cells were microinjected with C3 transferase, and subsequently treated with paclitaxel at anaphase onset, both pathways for actin redistribution were affected (Figure 7D, *n* = 5); specifically, delivery to the equatorial cortex from both the polar cortex and the midzone was impeded. Cells failed to divide long after entering anaphase (Figure 7D, 0–73 min), due to inhibition of both de novo assembly of nascent actin aggregates, and relocation of cortical actin filaments to the contractile ring.

## Discussion

### Polar Relaxation and Equatorial Stimulation Both Induce Contractile Ring Assembly and Cell Cleavage

By mechanically manipulating the spindles of double-labeled silkworm spermatocytes, we show how polar relaxation (as gauged by redistribution of cortical actin) and equatorial stimulation of the cortex (as gauged by de novo actin assembly and transport) both contribute to contractile ring assembly and furrow induction. We demonstrate that spindle microtubules deliver both inhibitory and stimulatory signals to the cell cortex during furrow formation, depending on their configuration and location within the cell. The dynamic astral microtubules drive actin filaments from the polar region to the equatorial cortex, which relaxes the polar cortex, while increasing cortical tension at the actin-dense equator. Meanwhile, bundles of interdigitating microtubules across the entire central spindle stimulate de novo assembly of actin aggregates at their overlapping plus ends, and deliver the aggregates to the equatorial cortex. These dual signaling mechanisms ensure that both cortical actin and central spindle-associated actin are delivered to the equatorial cortex, resulting in a robust contractile ring, and providing redundancy to safeguard an essential cellular process. Videos S4 and S9, as well as previous studies [34,35], provide hints that cytoplasmic actin filaments (perhaps vesicle bound; [29]) may constitute a third source of actin that could ultimately be delivered to the equatorial cortex by microtubules.

We show that in the absence of equatorial stimulation, contractile ring assembly and furrowing can still be induced by means of actin redistribution driven by cortical flow (Figure 3A and 3B and Figure 7C). We accomplished this by displacing the entire spindle apparatus, bearing “stimulatory” furrow cues that were reorganized (via spindle collapse), or C3 transferase-suppressed, to an arbitrary region of the cortex. The existence of a cortical flow of actin filaments away from the displaced spindle microtubules was established by our ability to intercept the flow locally, using a microneedle (Figure 3D). We found that the flow of cortical actin during cytokinesis can be induced by any microtubules positioned near the cortex, provided their plus ends are dynamic and accessible (i.e., neither interdigitating nor otherwise bundled or stabilized). We hypothesize that collapsing the spindle can mechanically free a subset of plus ends; this could allow those formerly interdigitating central spindle microtubules to become more dynamic and to take on properties held by astral microtubules, such as the ability to contact the cell cortex and induce cortical flow of actin filaments. Interdigitating microtubules in an intact central spindle would be able to contact the cortex only laterally, and as incorrectly oriented bundles, which should not be conducive for promoting cortical flow. As predicted by this finding, furrow formation can occur between a collapsed asterless spindle and its two detached asters in a remodeled cell (Figure 3C). We have also shown that this microtubule-driven actin flow begins shortly after anaphase onset and persists through telophase (Figure 3A and 3B), contributing to both contractile ring assembly at the equator and furrow inhibition at the poles throughout the furrow induction and ingression stages. Cortical flow during cytokinesis has previously been inferred or observed for contractile elements including myosin II [28,36,37] and pre-existing actin filaments [17,35,38], as well as for a membrane-bound receptor-ligand complex, membrane domains, and cell surface proteins [39–41]. As the movement of certain surface proteins mirrors that of cortical actin, it has been suggested that the actin network could serve as a scaffold for these proteins, thus engineering their co-transport to the furrow region [41].

How might microtubules interact with actin filaments to effect their exclusion from the poles? Since paclitaxel inhibits cortical flow (Figure 6B–6D), actin filaments are likely to be driven from the polar region towards the equatorial cortex by elongating microtubules. Following anaphase onset, the plus ends of astral microtubules can elongate toward and contact the cortex [20,42]. Such microtubules would be anchored to the spindle pole at their minus ends, and conceivably constrained at their plus ends by indirect attachment to a mobile cortical component, namely filamentous actin (analogous to cortical capture via Bim1/Kar9/Myo2 [43,44]). Given the microtubule's length, flexibility, geometrical orientation within the cell, and constraints on its direction of movement, its elongation may be sufficient to drive cortical actin toward the equator. Elongation of tethered microtubules against a fixed barrier can produce force of up to 10 pN, as gauged by microtubule buckling [45]. Presumably, this force could be harnessed to allow tip-attached cortical actin to “hitchhike” to the equatorial cortex, where it would be released, possibly by binding to myosin II [38] or anillin [46]. Alternatively, or in addition, actin filaments could be transported along the subset of microtubules that are contiguous to the cortex, and longitudinally aligned, with appropriate polarity. Motor proteins would provide the driving force for transport, with microtubule elongation serving only to extend the track toward the equator. Actin filaments could either be cargo for a plus-end–directed kinesin (attached via an adaptor protein), or else the actin could be coupled to the microtubule track via kinesin/myosinV-type hetero-motors (as modeled in [44,47]), and slide along the stationary microtubule toward the equatorial cortex, with kinesin providing directionality for the flow. The tracks could consist of the cortically apposed section of centrosome-attached astral microtubules and/or those released from the centrosomes [42,48–50] that become cortically apposed [34,47,51,52].

We also show that actin aggregates can be assembled de novo within the central spindle in the absence of cortical-flow based redistribution of actin, by using paclitaxel to impede the flow. Similarly, paclitaxel-stabilized microtubules can induce a cleavage furrow in mammalian cells [53], perhaps implying mechanistic conservation. We demonstrate that cortical stimulation can be induced at any region in the cortex by microtubule plus ends of the central spindle (Figure 6). Our data reveal that de novo–assembled cytoplasmic actin aggregates can comprise a supply of actin sufficient to form the contractile ring and induce cell cleavage (Figure 6D). Newly assembled actin aggregates are precursors of the contractile ring, since they ultimately merge with the ring (Figure 5). The existence of de novo assembly of actin during contractile ring formation is consistent with evidence from diverse cell types, including grasshopper spermatocytes (unpublished data), yeast [54,55], *Xenopus* eggs [56], and mammalian cells [17,38]; in addition, there is precedent for the presence of actin nucleating protein formin at microtubule plus ends [57]. Assembly of actin aggregates is sensitive to the Rho GTPase inhibitor, C3 transferase (Figure 6B, middle), supporting the existence of a microtubule-dependent zone of active RhoA during cleavage plane specification [5,23,58–62]. Our findings also raise the possibility that equatorial stimulation signals could be transported along stationary central spindle microtubule tracks from the spindle pole region to the midzone (Figure 5B), before ultimately reaching the equatorial cortex, perhaps via lateral transport.

Actin aggregates are assembled across the entire spindle midzone (Figure 5A and 5C); hence they must be transported laterally from the plus ends of the bundled microtubules to the equatorial cortex. We have captured this novel means of delivery using time-lapse sequences (Figures 5B, 6E, and 6F). Bundles of central spindle microtubules, some tipped with actin aggregates, splay laterally outward as the spindle changes shape, thereby conveying their cargo to the incipient furrow (Figure 5B). The actin cargo remains associated with the tips of the microtubule bundles until the bundles are within about 0.4 μm of the cortex, less than the length of the aggregate itself (Table 1). At this point, the microtubule-associated aggregates appear to be released from the microtubule bundles, in that the costaining at the microtubule tips disappears. The dissociation of the actin from the bundles may be mediated by kinesins bound to the bundle, as the aggregate appears to slide off the end of the bundle as it approaches the cortex (Table 1). This transport of actin aggregates may be largely independent of microtubule dynamics; when a post-anaphase, paclitaxel-stabilized spindle with mechanically exposed, bundled plus ends is brought close to the cortex by micromanipulation, it remains functional for delivery of actin aggregates to the cortex (Figure 6E and 6F). The induction of an ectopic furrow following cortical deposition of actin aggregates from microtubule plus ends (Figure 6D) indicates that actin-tipped microtubules may contain sufficient furrow constituents (i.e., structural constituents along with any co-transported stimulatory cues) to permit contractile ring assembly. This idea is consistent with work by Hu et al., showing that the tips of monopolar spindles contain critical stimulatory components (e.g., the Rho GEF Ect2) [21].

We propose that in the bipolar spindle, other spatially dynamic midzone components may also utilize this microtubule-based lateral transport mechanism—either co-transported with actin aggregates, or else delivered via a distinct set of midzone microtubules. By ensuring timely relocation of cytokinetic components, lateral transport could play a role in processes such as midzone signaling of the equatorial cortex, assembly and maintenance of the contractile ring and abscission. For example, the midzone component centralspindlin becomes localized to the equatorial cortex during anaphase. Bundled interdigitating microtubules, tipped with centralspindlin, may become docked at the contractile ring via Anillin [46,63]. We suggest that these tipped microtubules may originate from the central spindle and deliver their cargo to the cortex via lateral transport. Similarly, lateral transport could provide a means of delivery to the cortex for a recently characterized subset of vesicles. These vesicles are associated both with actin and with the central spindle, and are thought to contribute to furrow ingression [29]. In fixed cells, the authors saw thin microtubule bundles potentially connecting the central spindle with the periphery [29], consistent with a lateral transport mechanism. As another example, by early anaphase, the chromosomal passenger complex (CPC) is at the mitotic spindle midzone, whereas later in anaphase it is also present at the equatorial cortex (reviewed in [64]), perhaps arriving there by lateral transport. Interestingly, in *Dictyostelium*, the CPC component INCENP has been shown to bind actin [65]. It is also worth determining whether a subset of RhoA may reach the equatorial cortex via lateral transport, given that RhoA localization may depend on microtubule organization [58].

### Model for Microtubule Induction of Contractile Ring Assembly

**Figure 8:**
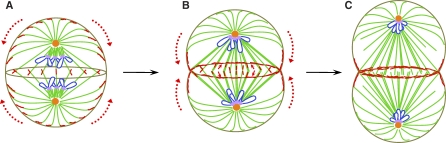
Model for Spindle Microtubule Induction of Contractile Ring Assembly (A) During furrow induction and ingression, dynamic astral microtubules exclude pre-existing actin filaments from the spindle pole (red dotted arrows). (B) This exclusion results in mass migration of filaments to the equator. Meanwhile, overlapping spindle microtubule plus ends at the equator promote de novo assembly of actin aggregates, which are delivered (small red arrows) to the equatorial cortex by splaying bundles of central spindle microtubules. (C) Actin filaments excluded from the polar region coalesce with actin aggregates transported from the central spindle to the equatorial cortex, for assembly of the contractile ring.

Our results explain how a single spindle component, the microtubule, can play opposing roles in two complementary pathways of contractile ring assembly. The seemingly incongruous stimulatory and inhibitory effects on the cortex are produced by different configurations of microtubules that populate different regions of the cytokinetic cell. We propose a microtubule induction model to describe how microtubules perform their dual signaling at the poles and the equator to induce contractile ring formation and cleavage furrow initiation. During early anaphase, dynamic astral microtubules at the poles inhibit ectopic furrow formation by excluding pre-existing actin filaments from the poles (Figure 8A, red dotted arrows and red lines at cortex), ultimately resulting in actin accumulation at the incipient equatorial furrow. Meanwhile, at the plus ends of the relatively stable central spindle microtubules, actin aggregates are assembled de novo, and enlarge over time (Figure 8A, red lines in midzone). As the cell progresses toward telophase, preexisting cortical actin filaments continue to flow from the polar cortex to the equator (Figure 8B, red dotted arrows). At the same time, the plus ends of bundled central spindle microtubules splay laterally to deliver the actin aggregates to the equatorial cortex (Figure 8B, red solid arrows). The newly delivered aggregates coalesce with the actin excluded from the polar cortex to assemble the contractile ring (Figure 8C).

In summary, we show that actin is redistributed into the contractile ring via two microtubule-dependent pathways that coexist during cytokinesis of the silkworm spermatocyte: equatorial stimulation and polar relaxation. These dual signaling pathways ensure the fidelity of cytokinesis, which fails only if both mechanisms are inhibited (Figure 7D). With some modification, our model could be generalized to multiple cell types. For example, cytokinetic cells of varying sizes and spindle geometries might rely more heavily on one mechanism than the other to achieve a global balance between tension and relaxation. We have demonstrated that the entire cell cortex is potentially responsive to both stimulatory and inhibitory cues from microtubules during cytokinesis in silkworm spermatocytes. How might the cell restrict the contractile ring to a localized band of equatorial cortex? By stimulating only the equatorial cortex while concomitantly relaxing other regions of the cortex, a cell could ensure cytokinetic fidelity by building in redundancy. Polar relaxation could be thought to include a component of equatorial stimulation in that pre-existing actin filaments are transported to the equatorial cortex. Equatorial stimulation, with its de novo actin assembly, recruits cytoplasmic G-actin to the equator. Hence, the seemingly disparate models ultimately result in redistribution of all available actin resources to the equator for ring assembly.

### Silkworm as a Model Organism

Our work with spermatocytes of the silkworm B. mori has enabled us to document a number of novel events essential for understanding contractile ring assembly. These spermatocytes are highly amenable to mechanical manipulations and imaging techniques, making them ideal for cytokinetic studies. Equally important, the silkworm genome has been sequenced [66], paving the way for molecular and genetic studies, such as RNAi inhibition of particular genes. Thus, we believe the silkworm spermatocyte has great potential as a model system for research in cell biology, cell physiology, and developmental biology—given its innate attributes coupled with the power of molecular genetics.

## Methods

### Primary cell culture.

The spermatocytes of silkworm Bombyx mori were isolated from the testes of a 5th instar larva and spread under inert halocarbon oil (400 oil, Halocarbon Products) to form a monolayer of cells in a glass chamber slide. Only cells in the first meiotic division were used.

### Microscopy.

Cells were observed with an inverted Zeiss Axiovert 135 microscope modified for both digital-enhanced polarization [6] and spinning disc confocal microscopy (CARV, Carl Zeiss). The microscope is equipped with a 1.4 NA achromatic-aplanatic condenser and a 1.45 NA/100X α-Plan objective (Carl Zeiss). An EM-CCD digital camera (Hamamatsu C9100–12), Simple PCI software (C-image), and Photoshop software (Adobe Systems) were used to record and process images. All images in a given category “*n*” are fully representative of all unshown data in that category.

### Micromanipulation.

Micromanipulation needles were pulled from glass tubing (outer diameter: 1.0 mm; inner diameter: 0.58 mm, World Precision Instruments) as described [67] using a microforge (Narishige, Model MF-830) and maneuvered with a Burleigh MIS-5000 series piezoelectric micromanipulator [6].

### Microinjection and preparation of injectants.

Glass tubing (outer diameter: 1.0 mm; inner diameter: 0.75 mm) with an internal capillary (World Precision Instruments) was pulled on a Flaming/Brown P-87 micropipette puller (Sutter Instrument Company) to produce micropipettes with a tip diameter ∼ 0.1 μm. The injectant was back loaded into the micropipette using a Hamilton syringe. Microinjection was conducted at 60 psi using a custom-built pneumatic injector maneuvered with a Burleigh MIS-5000 series piezoelectric micromanipulator.

*Alexa Fluor 568 tubulin*. Alexa Fluor 568 (Invitrogen) was conjugated to porcine tubulin (Cytoskeleton) following the protocol adapted from Peloquin et al. [68]. The labeled tubulin was resuspended in an injection buffer (20 mM PIPES, 0.25 mM MgSO_4_, 1 mM EGTA, 1 mM GTP, pH 6.8) to a final concentration of 3 mg/ml for microinjection.

*Phalloidin.* Alexa Fluor 488 phalloidin or rhodamine phalloidin (Invitrogen) in methanol was concentrated using a SpeedVac concentrator (Savant) and resuspended in the injection buffer to a final concentration of ∼6.6 μM for microinjection. Although the intracellular concentration of phalloidin was low, controls were performed to ensure that the phalloidin did not adversely affect the function of the actin filaments. The timing of cell division was not slowed down in a phalloidin-treated cell relative to uninjected controls (as in Table S1).

*C3 transferase.* C3 transferase (Cytoskeleton) was stored as 1 mg/ml aliquots at −70 °C in a buffer containing 500 mM imidazole (pH 7.5), 50 mM Tris HCl (pH 7.5), 1.0 mM MgCl_2_, 200 mM NaCl, 5% sucrose, and 1% dextran. Immediately before microinjection, the C3 transferase was mixed with Alexa Fluor 568 tubulin and Alexa Fluor 488 phalloidin to a final concentration of 0.5 mg/ml in the micropipette.

### Live cell labeling with the tubulin tracker and Hoechst stain.

Tubulin Tracker (Invitrogen) was used as directed, with the Oregon Green 488 Taxol (paclitaxel) diluted to 50 μM in insect Ringer's solution. The diluted Tubulin Tracker was micropipetted around the target cells, resulting in a further dilution of at least 1000 fold. Hoechst 33342 (Invitrogen) was stored as 10 mg/ml stock aliquots at −20 °C and diluted in Tubulin Tracker buffer to 0.5 mg/ml before micropipetting.

###  Immunofluorescence microscopy.

Silkworm spermatocytes were fixed and stained as previously described [6]. Microtubules were stained with tubulin primary antibody (Chemicon) and Alexa Fluor 488 conjugated secondary antibody (Invitrogen); actin filaments, with rhodamine phalloidin (Invitrogen); and chromosomes, with DAPI.

### Labeling color scheme.

In all figures, microtubules are shown in green, and actin in red (false colored, if necessary). Chromosomes, when labeled, are blue (Hoechst for live cells, DAPI for fixed).

### Measurement of cortical stiffness.

The degree of stiffness at one region of the spermatocyte cortex relative to another region was assayed as follows. The spindle of a cell in anaphase was collapsed (by pushing the spindle poles together) and mechanically repositioned near the cortex to induce cortical flow of actin filaments. After waiting 5–10 min to allow for redistribution of actin, the first region of interest was probed with the side of a flexible microneedle, tangential to the cortex. Depending on the flexural rigidity of the needle and the local cortical stiffness, a given needle would either deform the cortex by flattening it, without deflecting the needle, or the cortex would bend the needle. The cell was repositioned (by rotating the microscope slide by 180°) to bring the second region of interest in proximity with the needle, and then reprobed.

## Supporting Information

Table S1 Summary of Experimental Design and Results (230 KB DOC)

Video S1 Cytokinesis of a Silkworm Spermatocyte as Seen by Polarization Microscopy The birefringence of spindle microtubules is shown with dark compensation. Video S1 corresponds to Figure 1A.
(8.66 MB AVI)

Video S2 Cytokinesis of a Silkworm Spermatocyte with Labeled Actin and Microtubules as Seen by Confocal Microscopy Confocal time-lapse imaging of the cytokinesis of a silkworm spermatocyte microinjected with rhodamine tubulin (microtubules false colored green) and low-level Alexa Fluor 488 phalloidin (actin false colored red). Actin aggregates appeared at the microtubule plus ends during early anaphase, and then fused with the contractile ring that bisected the cell. Video S2 corresponds to Figure 1B.
(5.75 MB AVI)

Video S3 Cortical Flow of Actin Induced by Asymmetrically Distributed Asters during Anaphase Cortical actin filaments were excluded from the polar region occupied by asymmetrically distributed asters in early anaphase. Cells in Video S3 labeled as in Video S2. Video S3 corresponds to Figure 2A.
(1.28 MB AVI)

Video S4 Cortical Flow of Actin Induced by Asymmetrically Distributed Asters Results in Furrow Initiation during Anaphase Cortical actin filaments were excluded from the polar region occupied by asymmetrically distributed asters during anaphase. The excluded actin assembled a contractile ring around the equator of the naturally shifted spindle. The two asters were in different focal planes. In frames ∼1–13 (especially frames 6, 7, 9, and 10), actin filaments appeared to be radiating out from the microtubule aster, parallel to and possibly overlapping the astral microtubules. We hypothesize that this may be part of a mechanism by which cytoplasmic actin filaments are cleared from the polar region. These actin asters can also be seen in Video S9. Cells in Video S4 labeled as in Video S2. Video S4 corresponds to Figure 2B.
(3.15 MB AVI)

Video S5 Cortical Flow of Actin Driven by Microtubules of a Collapsed Spindle during Anaphase The redistribution of actin filaments resulted in asymmetric cell division. Cells in Video S5 labeled as in Video S2. Video S5 corresponds to Figure 3A.
(1.95 MB AVI)

Video S6 Cortical Flow of Actin Driven by Microtubules of a Collapsed Spindle during Telophase The redistribution of actin filaments resulted in asymmetric cell division. Cells in Video S6 labeled as in Video S2. Video S6 corresponds to Figure 3B.
(2.99 MB AVI)

Video S7 A Contractile Ring Formed between a Scrambled Spindle and Two Asters We hypothesize that ring formation was due to actin exclusion by microtubules from both structures. Cell in Video S7 labeled as in Video S2. Video S7 corresponds to Figure 3C.
(2.04 MB AVI)

Video S8 Induced Cortical Flow of Actin Could Be Intercepted Using a Microneedle The spindle was collapsed and pushed close to the cell's upper cortex to induce actin flow. The microneedle (arrow on first frame), which indented but did not pierce the plasma membrane, locally intercepted the actin flow, with brighter fluorescence accumulating on the side of the needle facing the repositioned spindle. Cell in Video S8 labeled as in Video S2. Video S8 corresponds to Figure 3D.
(2.82 MB AVI)

Video S9 De Novo Assembly of Actin Aggregates at Overlapping Microtubule Plus Ends in the Central Spindle Cell in Video S9 labeled as in Video S2. Video S9 corresponds to Figure 5A.
(3.33 MB AVI)

Video S10 Delivery of Actin Aggregates into the Ingressing Furrow by a Splaying Microtubule Bundle The splaying microtubule bundle is marked by an arrow on the first frame. Cell in Video S10 labeled as in Video S2. Video S10 corresponds to Figure 5B.
(1.97 MB AVI)

Video S11 De Novo Assembly of Actin Aggregates Was Not Dependent on Microtubule Dynamics Compare Video S11 to Videos S2 and S9. Microtubules were labeled and stabilized by Oregon green paclitaxel (green), and actin filaments were labeled by rhodamine phalloidin (red). Video S11 corresponds to Figure 5C.
(1.86 MB AVI)

Video S12 Cortical Flow of Actin Was Dependent on Microtubule Dynamics Neither the stabilized lateral spindle nor the evolving monopolar spindle induced unidirectional flow of actin filaments (compare to Videos S5–S8). Cell in Video S12 labeled as in Video S11. Video S12 corresponds to Figure 6B.
(7.03 MB AVI)

Video S13 Delivery of Actin Aggregates from the Tips of the Stabilized Microtubule Bundles to the Cortex Actin aggregates were assembled at the plus ends of stabilized microtubule bundles from a lateral spindle, and were delivered to the non-equatorial cortex where splaying microtubule bundles were in contact with the cortex (cell's lower left cortex). Cell in Video S13 labeled as in Video S11. Video S13 corresponds to Figure 6C.
(5.21 MB AVI)

Video S14 Delivery of an Actin Aggregate from the Tip of a Microtubule Bundle to the Cortex The microtubule bundle is boxed in the first frame of the video. The remodeled spindle had chromosomes in its center and microtubule plus ends pointing outward. Cell in Video S14 labeled as in Video S11. Video S14 corresponds to Figure 6E.
(1.62 MB AVI)

Video S15 A 3D View of a Giant Aster, Showing Actin-Tipped Bundles A stack of 2D images of the cell in Figure 6E was reprocessed to create a 3D image, by rotation around the *y*-axis. Microtubules (green) were stabilized and labeled with Oregon Green Paclitaxel, and actin (red) was labeled using low-level rhodamine phalloidin. The giant aster developed from a post anaphase spindle, which was collapsed by a microneedle. Microtubule bundles in the aster were oriented with their plus ends closest to the cortex, and many of them were tipped with actin aggregates. A subset of these actin aggregates were ultimately delivered to and merged into the cortex after the aster was maneuvered to a location near the cortex. Video S15 corresponds to Figure 6E.
(924 KB AVI)

Video S16 A 3D View of a Bipolar Spindle, Showing Overlapping Actin-Tipped Bundles in the Central Spindle A stack of 2D images of an anaphase cell (at a stage similar to that of the cell in Figure 1B) was reprocessed to create a 3D image, by rotation first around the *y*-axis, and then around the *x*-axis. Microtubules (green) were stabilized and labeled with Oregon Green Paclitaxel, actin (red) was labeled by low-level rhodamine phalloidin, and the nucleus (blue) was stained with Hoechst. Microtubule bundles from the two half spindles radiated outwards to the equatorial cortex, displaying tips coated with actin aggregates. Such actin coated microtubule bundles were absent on the side of the cell facing the coverslip (see the vertical rotation). Presumably because the cell was flatter on that side, the bundles had to travel a shorter distance to deliver their cargo; hence, they may have reached the cortex sooner than the bundles on the opposite side. In this cell, the chromosomes were already segregated to the poles, and some of the actin aggregates had already been delivered to the equatorial cortex by the microtubule plus ends; thus, actin aggregates could no longer be seen in the center of the spindle. Two pairs of centrosomes at the poles of the cell were clearly visible.
(2.43 MB AVI)

## References

[pbio-0060209-b001] D'Avino PP, Savoian MS, Glover DM (2005). Cleavage furrow formation and ingression during animal cytokinesis: a microtubule legacy. J Cell Sci.

[pbio-0060209-b002] Burgess DR, Chang F (2005). Site selection for the cleavage furrow at cytokinesis. Trends Cell Biol.

[pbio-0060209-b003] Eggert US, Mitchison TJ, Field CM (2006). Animal cytokinesis: from parts list to mechanisms. Annu Rev Biochem.

[pbio-0060209-b004] Glotzer M (2004). Cleavage furrow positioning. J Cell Biol.

[pbio-0060209-b005] Piekny A, Werner M, Glotzer M (2005). Cytokinesis: welcome to the Rho zone. Trends Cell Biol.

[pbio-0060209-b006] Alsop GB, Zhang D (2003). Microtubules are the only structural constituent of the spindle apparatus required for induction of cell cleavage. J Cell Biol.

[pbio-0060209-b007] Canman JC, Wells WA (2004). Rappaport furrows on our minds: the ASCB Cytokinesis Meeting Burlington, VT July 22–25, 2004. J Cell Biol.

[pbio-0060209-b008] Wolpert L (1960). The mechanics and mechanism of cleavage. Int Rev Cytol.

[pbio-0060209-b009] White JG, Borisy GG (1983). On the mechanisms of cytokinesis in animal cells. J Theor Biol.

[pbio-0060209-b010] Yoshigaki T (1999). Simulation of density gradients of astral microtubules at cell surface in cytokinesis of sea urchin eggs. J Theor Biol.

[pbio-0060209-b011] Hiramoto Y (1990). Mechanical properties of the cortex before and during cleavage. Ann N Y Acad Sci.

[pbio-0060209-b012] Schroeder TE (1981). The origin of cleavage forces in dividing eggs. A mechanism in two steps. Exp Cell Res.

[pbio-0060209-b013] Canman JC, Hoffman DB, Salmon ED (2000). The role of pre- and post-anaphase microtubules in the cytokinesis phase of the cell cycle. Curr Biol.

[pbio-0060209-b014] Hird SN, White JG (1993). Cortical and cytoplasmic flow polarity in early embryonic cells of Caenorhabditis elegans. J Cell Biol.

[pbio-0060209-b015] Kurz T, Pintard L, Willis JH, Hamill DR, Gonczy P (2002). Cytoskeletal regulation by the Nedd8 ubiquitin-like protein modification pathway. Science.

[pbio-0060209-b016] Krendel M, Zenke FT, Bokoch GM (2002). Nucleotide exchange factor GEF-H1 mediates cross-talk between microtubules and the actin cytoskeleton. Nat Cell Biol.

[pbio-0060209-b017] Zhou M, Wang YL (2008). Distinct pathways for the early recruitment of myosin II and actin to the cytokinetic furrow. Mol Biol Cell.

[pbio-0060209-b018] Rappaport R (1961). Experiments concerning the cleavage stimulus in sand dollar eggs. J Exp Zool.

[pbio-0060209-b019] Rieder CL, Khodjakov A, Paliulis LV, Fortier TM, Cole RW (1997). Mitosis in vertebrate somatic cells with two spindles: implications for the metaphase/anaphase transition checkpoint and cleavage. Proc Natl Acad Sci U S A.

[pbio-0060209-b020] Canman JC, Cameron LA, Maddox PS, Straight A, Tirnauer JS (2003). Determining the position of the cell division plane. Nature.

[pbio-0060209-b021] Hu CK, Coughlin M, Field CM, Mitchison TJ (2008). Cell polarization during monopolar cytokinesis. J Cell Biol.

[pbio-0060209-b022] Cao LG, Wang YL (1996). Signals from the spindle midzone are required for the stimulation of cytokinesis in cultured epithelial cells. Mol Biol Cell.

[pbio-0060209-b023] Bement WM, Benink HA, von Dassow G (2005). A microtubule-dependent zone of active RhoA during cleavage plane specification. J Cell Biol.

[pbio-0060209-b024] Saint R, Somers WG (2003). Animal cell division: a fellowship of the double ring. J Cell Sci.

[pbio-0060209-b025] Bringmann H, Cowan CR, Kong J, Hyman AA (2007). LET-99, GOA-1/GPA-16, and GPR-1/2 are required for aster-positioned cytokinesis. Curr Biol.

[pbio-0060209-b026] Bringmann H, Hyman AA (2005). A cytokinesis furrow is positioned by two consecutive signals. Nature.

[pbio-0060209-b027] Motegi F, Velarde NV, Piano F, Sugimoto A (2006). Two phases of astral microtubule activity during cytokinesis in C. elegans embryos. Dev Cell.

[pbio-0060209-b028] Werner M, Munro E, Glotzer M (2007). Astral signals spatially bias cortical myosin recruitment to break symmetry and promote cytokinesis. Curr Biol.

[pbio-0060209-b029] Albertson R, Cao J, Hsieh TS, Sullivan W (2008). Vesicles and actin are targeted to the cleavage furrow via furrow microtubules and the central spindle. J Cell Biol.

[pbio-0060209-b030] Bonaccorsi S, Giansanti MG, Gatti M (1998). Spindle self-organization and cytokinesis during male meiosis in asterless mutants of Drosophila melanogaster. J Cell Biol.

[pbio-0060209-b031] Yamashiki N, Kawamura N (1998). Behavior of centrioles during meiosis in the male silkworm, Bombyx mori (Lepidoptera). Dev Growth Differ.

[pbio-0060209-b032] Alsop GB, Zhang D (2004). Microtubules continuously dictate distribution of actin filaments and positioning of cell cleavage in grasshopper spermatocytes. J Cell Sci.

[pbio-0060209-b033] Kishi K, Sasaki T, Kuroda S, Itoh T, Takai Y (1993). Regulation of cytoplasmic division of Xenopus embryo by rho p21 and its inhibitory GDP/GTP exchange protein (rho GDI). J Cell Biol.

[pbio-0060209-b034] Foe VE, Field CM, Odell GM (2000). Microtubules and mitotic cycle phase modulate spatiotemporal distributions of F-actin and myosin II in Drosophila syncytial blastoderm embryos. Development.

[pbio-0060209-b035] Cao LG, Wang YL (1990). Mechanism of the formation of contractile ring in dividing cultured animal cells. II. Cortical movement of microinjected actin filaments. J Cell Biol.

[pbio-0060209-b036] DeBiasio RL, LaRocca GM, Post PL, Taylor DL (1996). Myosin II transport, organization, and phosphorylation: evidence for cortical flow/solation-contraction coupling during cytokinesis and cell locomotion. Mol Biol Cell.

[pbio-0060209-b037] Yumura S (2001). Myosin II dynamics and cortical flow during contractile ring formation in Dictyostelium cells. J Cell Biol.

[pbio-0060209-b038] Murthy K, Wadsworth P (2005). Myosin-II-dependent localization and dynamics of F-actin during cytokinesis. Curr Biol.

[pbio-0060209-b039] Koppel DE, Oliver JM, Berlin RD (1982). Surface functions during mitosis. III. Quantitative analysis of ligand-receptor movement into the cleavage furrow: diffusion vs. flow. J Cell Biol.

[pbio-0060209-b040] Ng MM, Chang F, Burgess DR (2005). Movement of membrane domains and requirement of membrane signaling molecules for cytokinesis. Dev Cell.

[pbio-0060209-b041] Bauer T, Motosugi N, Miura K, Sabe H, Hiiragi T (2008). Dynamic rearrangement of surface proteins is essential for cytokinesis. Genesis.

[pbio-0060209-b042] Rusan NM, Wadsworth P (2005). Centrosome fragments and microtubules are transported asymmetrically away from division plane in anaphase. J Cell Biol.

[pbio-0060209-b043] Huisman SM, Segal M (2005). Cortical capture of microtubules and spindle polarity in budding yeast - where's the catch. J Cell Sci.

[pbio-0060209-b044] Rodriguez OC, Schaefer AW, Mandato CA, Forscher P, Bement WM (2003). Conserved microtubule-actin interactions in cell movement and morphogenesis. Nat Cell Biol.

[pbio-0060209-b045] Janson ME, de Dood ME, Dogterom M (2003). Dynamic instability of microtubules is regulated by force. J Cell Biol.

[pbio-0060209-b046] Gregory SL, Ebrahimi S, Milverton J, Jones WM, Bejsovec A (2008). Cell division requires a direct link between microtubule-bound RacGAP and Anillin in the contractile ring. Curr Biol.

[pbio-0060209-b047] Goode BL, Drubin DG, Barnes G (2000). Functional cooperation between the microtubule and actin cytoskeletons. Curr Opin Cell Biol.

[pbio-0060209-b048] Keating TJ, Peloquin JG, Rodionov VI, Momcilovic D, Borisy GG (1997). Microtubule release from the centrosome. Proc Natl Acad Sci U S A.

[pbio-0060209-b049] Zimmerman WC, Sillibourne J, Rosa J, Doxsey SJ (2004). Mitosis-specific anchoring of gamma tubulin complexes by pericentrin controls spindle organization and mitotic entry. Mol Biol Cell.

[pbio-0060209-b050] Waterman-Storer C, Duey DY, Weber KL, Keech J, Cheney RE (2000). Microtubules remodel actomyosin networks in Xenopus egg extracts via two mechanisms of F-actin transport. J Cell Biol.

[pbio-0060209-b051] Gavin RH (1997). Microtubule-microfilament synergy in the cytoskeleton. Int Rev Cytol.

[pbio-0060209-b052] Sider JR, Mandato CA, Weber KL, Zandy AJ, Beach D (1999). Direct observation of microtubule-f-actin interaction in cell free lysates. J Cell Sci.

[pbio-0060209-b053] Shannon KB, Canman JC, Ben Moree C, Tirnauer JS, Salmon ED (2005). Taxol-stabilized microtubules can position the cytokinetic furrow in mammalian cells. Mol Biol Cell.

[pbio-0060209-b054] Yonetani A, Lustig RJ, Moseley JB, Takeda T, Goode BL (2008). Regulation and targeting of the fission yeast formin cdc12p in cytokinesis. Mol Biol Cell.

[pbio-0060209-b055] Vavylonis D, Wu JQ, Hao S, O'Shaughnessy B, Pollard TD (2008). Assembly mechanism of the contractile ring for cytokinesis by fission yeast. Science.

[pbio-0060209-b056] Noguchi T, Mabuchi I (2001). Reorganization of actin cytoskeleton at the growing end of the cleavage furrow of Xenopus egg during cytokinesis. J Cell Sci.

[pbio-0060209-b057] Feierbach B, Verde F, Chang F (2004). Regulation of a formin complex by the microtubule plus end protein tea1p. J Cell Biol.

[pbio-0060209-b058] Nishimura Y, Yonemura S (2006). Centralspindlin regulates ECT2 and RhoA accumulation at the equatorial cortex during cytokinesis. J Cell Sci.

[pbio-0060209-b059] Somers WG, Saint R (2003). A RhoGEF and Rho family GTPase-activating protein complex links the contractile ring to cortical microtubules at the onset of cytokinesis. Dev Cell.

[pbio-0060209-b060] Yuce O, Piekny A, Glotzer M (2005). An ECT2-centralspindlin complex regulates the localization and function of RhoA. J Cell Biol.

[pbio-0060209-b061] Zavortink M, Contreras N, Addy T, Bejsovec A, Saint R (2005). Tum/RacGAP50C provides a critical link between anaphase microtubules and the assembly of the contractile ring in Drosophila melanogaster. J Cell Sci.

[pbio-0060209-b062] Zhao WM, Fang G (2005). MgcRacGAP controls the assembly of the contractile ring and the initiation of cytokinesis. Proc Natl Acad Sci U S A.

[pbio-0060209-b063] D'Avino PP, Takeda T, Capalbo L, Zhang W, Lilley KS (2008). Interaction between Anillin and RacGAP50C connects the actomyosin contractile ring with spindle microtubules at the cell division site. J Cell Sci.

[pbio-0060209-b064] Ruchaud S, Carmena M, Earnshaw WC (2007). Chromosomal passengers: conducting cell division. Nat Rev Mol Cell Biol.

[pbio-0060209-b065] Chen Q, Lakshmikanth GS, Spudich JA, De Lozanne A (2007). The localization of inner centromeric protein (INCENP) at the cleavage furrow is dependent on Kif12 and involves interactions of the N terminus of INCENP with the actin cytoskeleton. Mol Biol Cell.

[pbio-0060209-b066] Xia Q, Zhou Z, Lu C, Cheng D, Dai F (2004). A draft sequence for the genome of the domesticated silkworm (Bombyx mori). Science.

[pbio-0060209-b067] Zhang D, Nicklas RB (1999). Micromanipulation of chromosomes and spindles in insect spermatocytes. Methods Cell Biol.

[pbio-0060209-b068] Peloquin J, Komarova Y, Borisy G (2005). Conjugation of fluorophores to tubulin. Nat Methods.

