# Supplementary material for: Redundant Mechanisms Recruit Actin into the Contractile Ring in Silkworm Spermatocytes
Source: PLoS Biol. 2008 Sep 2;6(9):e209. doi: 10.1371/journal.pbio.0060209 (PMC2528054; doi:10.1371/journal.pbio.0060209)
Supplement: Supplementary file 1 [file pbio.0060209.st001.doc]

| **Supplemental Table 1 – SUMMARY OF TREATMENTS** | | | | | |
| --- | --- | --- | --- | --- | --- |
| **Fig.** | **Panel** | **Video** | **Treatments** | **“n”** | **Results/Comments** |
| **1** | A | S1 | Uninjected control; Polarization Microscopy. | 9 | Anaphase-onset to cleavage: 48.4 ± 2.5 min. |
|  | B | S2 | Phalloidin- and tubulin-injected, double-labeled control; Confocal Microscopy. | 4 | Anaphase-onset to cleavage: 49.5 ± 5.0 min.  Timing of division not impeded by low levels of Phalloidin. |
|  | C, a-c |  | Fixed in metaphase/anaphase; immunolabeled. | 15 | Astral flagella visible. |
|  | C, d-e |  | Live cells injected with phalloidin and tubulin. | 15 | Naturally dislocated asters visible. |
| **2** | A | S3 | Asters naturally on same side of spindle; labeled as in 1B. | 4 | Actin exclusion by asters (i.e., cortical flow) prior to furrow initiation. Contractile ring assembly not imaged. |
|  | B | S4 | Asters naturally on same side of spindle; labeled as in 1B. | 6 | Asymmetric assembly of contractile ring following actin exclusion. |
| **3** | A | S5 | Anaphase spindle collapsed/repositioned; labeled as in 1B. | 12 | Cortical actin was induced to flow away from repositioned spindle. |
|  | B | S6 | Telophase spindle collapsed/repositioned; labeled as in 1B. | 26 | Cortical flow of actin was induced; original furrow regressed. |
|  | C | S7 | Anaphase spindle collapsed/repositioned; separated from detached asters; labeled as in 1B. | 8 | Furrowing occurred between collapsed spindle and detached asters. |
|  | D | S8 | Anaphase spindle collapsed/repositioned, cortex indented by needle; labeled as in 1B. | 5 | Cortical flow of actin was induced; needle intercepted flow. |
| **4** | A, B |  | Probed actin-dense (A) and actin-poor (B) regions of cortex, following induction of flow, as in 3A; Polarization Microscopy. | 7 | Actin-dense region of cortex was stiffer than actin-poor region. |
| **5** | A | S9 | Cell labeled as in 1B. | 15 | Emergence of actin aggregates over time. |
|  | B | S10 | Cell labeled as in 1B. | 13 | Delivery of actin aggregates to cortex. |
|  | C | S11 | Oregon Green paclitaxel labeled/stabilized microtubules; injected with phalloidin. | 11 | Assembly of actin aggregates not inhibited by paclitaxel. |
| **6** | B | S12 | Manipulated to form lateral – monopolar spindle; labeled as in 5C. | 4 | Stabilized spindle could deliver actin aggregates. |
|  | C | S13 | Manipulated to form stabilized lateral spindle; labeled as in 5C. | 12 | Stabilized lateral spindle could deliver actin aggregates. |
|  | D | S14 | Manipulated to form monopolar spindle; labeled as in 5C. | 6 | Stabilized monopolar spindle could deliver actin aggregates. |
|  | E, F | S15 | Manipulated to form giant aster; labeled as in 5C. | 7 | Stabilized giant aster could deliver actin aggregates. |
| **7** | A |  | Control, labeled as in 1B. | 15 | Actin present within central spindle and at equatorial cortex. |
|  | B |  | C3 transferase; labeled as in 1B. | 9 | No actin aggregates in central spindle. |
|  | C |  | C3 transferase plus MT relocation; labeled as in 1B. | 8 | Cortical flow of actin could still be induced. |
|  | D |  | C3 transferase plus paclitaxel; labeled as in 5C. | 5 | Furrow failed to initiate; no cortical flow or actin aggregates. |
